# Panretinal Photocoagulation Using Short-Pulse Laser Induces Less Inflammation and Macular Thickening in Patients with Diabetic Retinopathy

**DOI:** 10.1155/2017/8530261

**Published:** 2017-07-06

**Authors:** Yoshihiro Takamura, Shogo Arimura, Seiji Miyake, Takehiro Matsumura, Makoto Gozawa, Kentaro Iwasaki, Masaru Inatani

**Affiliations:** Department of Ophthalmology, Faculty of Medical Sciences, University of Fukui, Eiheiji-cho, Yoshida-gun, Fukui-ken 910-1193, Japan

## Abstract

We compared the effect of panretinal photocoagulation (PRP) using short-pulse laser (SPL) and conventional laser, regardless of the number of spots, in terms of their effect on the progression of diabetic macular edema (DME) and anterior flare intensity (AFI) in patients with high-risk nonproliferative diabetic retinopathy (non-PDR). Forty-two eyes of 42 patients were subjected to PRP using the conventional argon laser (Conv group) or SPL (SPL group). CRT and AFI levels in the SPL group were significantly lower than those in the Conv group (CRT at 4, 6, and 10 weeks; AFI at 6, 10, and 18 weeks). Eyes of rabbits were photocoagulated using conventional laser with 500 spots (Conv 500s), SPL with 500 spots (SPL 500s), or 1000 spots (SPL 1000s). Vascular endothelial growth factor (VEGF), interleukin-6 (IL-6), intercellular adhesion molecule-1 (ICAM-1), and monocyte chemotactic protein-1 (MCP-1) levels in vitreous humor were measured using an immunoassay. Compared to conventional laser, VEGF, IL-6, and MCP-1 levels were significantly lower in the SPL 1000s and SPL 500s groups. In patients with high-risk non-PDR, SPL has a greater preventive effect on the progression of DME and AFI and produces less inflammatory cytokines than conventional lasers.

## 1. Introduction

Retinal laser photocoagulation is recognized as a standard treatment of proliferative diabetic retinopathy (PDR) [1, 2]. Panretinal photocoagulation (PRP) for the treatment of ischemic lesions involves the purposeful destruction of a fraction of the photoreceptors, as well as other more superficial retinal layers [3]. Although PRP contributes to decrease the risks of visual disturbance in the patients with severe non-PDR (NPDR) and PDR, the progression of diabetic macular edema (DME) sometimes occurs after PRP.

Some reports indicate that the elevated levels of cytokines and inflammatory reactions may be involved in the pathogenesis of the macular edema after PRP [4–7]. Apart from our study group, other investigators have also showed that thermal burns from laser photocoagulation induce the upregulation of vascular endothelial growth factor (VEGF) and several proinflammatory cytokines in mice and rabbits [7, 8]. In addition, it has been reported that the vitreous humor levels of cytokines, including VEGF, interleukin-6 (IL-6), intercellular adhesion molecule-1 (ICAM-1), and monocyte chemotactic protein-1 (MCP-1), are related to retinal permeability and to DME severity [9]. Thus, it is suggested that inflammation plays an important role in the progression of DME after PRP.

Recently, the pattern scan laser system (PASCAL® Streamline, Topcon Medical Laser systems, Santa Clara, CA, USA) was developed as a novel semiautomatic photocoagulator that delivers single applications of multiple laser burns in a shorter pulse duration of 10–30 ms [10]. This shorter pulse duration results in the destruction within the retina and choroid and is less painful and offers better preservation of retinal sensitivity for the patient than the conventional laser [11–14]. Ito et al. showed that short-pulse laser (SPL) induced fewer inflammatory cytokines than the conventional-pulse-duration laser in the retina of mice [7]. However, the SPL requires a greater number of spots to complete PRP than conventional laser because the expansion of laser scars of SPL is less [12, 15]. Chappelow et al. reported that the same number of spots was less effective in SPL-treated eyes than in conventional laser-treated eyes to prevent the progression of the neovascularization in the patients with high-risk PDR [16].

The increased number of spots would induce higher intraocular inflammatory cytokine levels, and it may lead to more severe inflammation and worsening of DME after PRP, even in the setting of SPL. Thus, it is still questionable whether SPL with greater number of shots is a less-invasive treatment than conventional lasers for high-risk NPDR or PDR. In this study, we compared the clinical effects PRP using SPL versus conventional lasers in patients with high-risk NPDR, using macular thickness and the anterior flare intensity (AFI) as indicators of anterior inflammation. In addition, we performed photocoagulation in the eyes of pigmented rabbits using conventional lasers or SPL with the same or twice the number of spots and compared the amount of inflammatory cytokines in the vitreous humor.

## 2. Method

### 2.1. Clinical Study

This retrospective comparative study adhered to the tenets of the Declaration of Helsinki and was approved by the University of Fukui Institutional Review Board. Consecutive patients with high-risk PDR underwent four-session PRP using a conventional laser or a SPL between May 2013 and October 2014. The study inclusion criteria included the following: (1) age > 20 years, (2) newly diagnosed very severe NPDR or PDR, (3) no history or clinical evidence of prior PRP, (4) subsequent PRP treatment using the conventional argon laser or SPL, (5) follow-up for ≥6 months, and (6) fluorescein angiography and spectral domain optical coherence tomography (SD-OCT) performed within 2 weeks before PRP. Severe NPDR was diagnosed if one or more quadrant has intraretinal microvascular abnormalities (IRMA), two quadrants or more have venous beading, or four quadrants have 20 retinal hemorrhages. PDR was defined as neovascularization of the disc (NVD) or elsewhere (NVE), or vitreous or preretinal hemorrhage. The exclusion criteria included the following: (1) other retinal disease such as retinal vein occlusion or uveitis, (2) a history of the cataract surgery within 12 months and any other intraocular surgeries including the vitrectomy at any time point before PRP, (3) a history of other intraocular treatment such as intravitreal injection, and (4) simultaneous focal/grid laser photocoagulation, (5) intraocular pressure > 22 mm Hg, (6) use of topical medications containing prostaglandin derivatives, and (7) vitreous hemorrhage. A total of 21 consecutive eyes were identified. We then identified an equal number of consecutive eyes using the same inclusion criteria, except that PRP was performed using a conventional argon laser with either direct ophthalmoscopy before acquisition of the pattern scan photocoagulator, PASCAL (April 2013 or earlier). The patients who underwent PRP using the conventional argon laser and PASCAL were defined as the Conv group and the SPL group, respectively.

All PRP procedures were performed in a darkened room approximately 30 minutes after the eye was pharmacologically dilated with 1% tropicamide and 2.5% phenylephrine. All eyes were anesthetized with topical proparacaine drops. The same clinician (T.T.) performed all single-session PRP procedures. The patients opted to divide the treatment into four sessions, and PRP was performed four times, with each session 2 weeks apart from the previous one, in retinal lesions around the vascular arcades as far anterior as possible beyond the equator with Super Quad 160 contact lens (Volk Optical, Inc., Mentor, OH, USA).

For the conventional argon group (Conv group), a retinal photocoagulator MC-300 (NIDEK, Co. Ltd., Aichi, Japan) was used under the following conditions: (1) the laser power set to 200 mW, increased by 10–20 mW until a gray/white lesion was attained, the duration of exposure was 200 ms in yellow wavelength (577 nm), (2) the coagulation spot size was adjusted to 200 *μ*m with a space the size of approximately one coagulation spot between each of the spots.

SPL was used with the PASCAL photocoagulator (Optimedica Corporation, Santa Barbara, CA, USA), which uses a frequency-doubled neodymium-doped yttrium aluminum garnet solid-state laser with a wavelength of 577 nm. The laser parameters were as follows: (1) spot size of 200 *μ*m, (2) pulse duration of 20 ms, (3) type of laser spot 5 × 5 and 4 × 4 multispot arrays, (4) burn intensity of 300 mW, increased until a gray/white lesion was attained, and (5) spacing of 500 *μ*m.

All patients who had recurrence or persistence of neovascularization were instructed to receive salvage treatment, including additional laser, intravitreal injection of anti-VEGF drug, or vitrectomy surgery.

All patients underwent an examination that included best-corrected visual acuity (BCVA) measurement (Snellen), slit-lamp examination, dilated fundus examination, fundus photography, and SD-OCT before (baseline) and 2, 4, 6, 10, and 18 weeks after PRP. BCVA measured with a Landolt chart was converted to logarithm of the minimum angle of resolution (logMAR).

The primary objective of this study was to compare the mean average of AFI and central retinal thickness (CRT) between the Conv group and the SPL group. AFI was measured using a laser flare cell meter (FC-1000, Kowa Co. Ltd., Nagoya, Japan) before (baseline) and at 2, 4, 6, 10, and 18 weeks after PRP. Ten measurements were taken 30 min after the application of 0.5% tropicamide and 0.5% phenylephrine hydrochloride (Mydrin P, Santen) and averaged to obtain the final results for flare intensity. CRT was measured at the same time points using SD-OCT (Cirrus OCT, Carl Zeiss Meditec, Dublin, CA). To minimize measurement error, all tests were performed by the same experienced examiner (S.A.). This examiner was masked to treatment status, including the procedure of PRP using argon or PASCAL and the duration after PRP. Secondary outcome measures included incidence of postlaser vitreous hemorrhage, neovascularization of the iris (NVI), neovascular glaucoma (NVG), and requirement for the additional laser and pars plana vitrectomy.

## 3. Animal Study

### 3.1. Animals

All animal experiments were approved by the University of Fukui Institutional Animal Care Committees and were conducted in accordance with the Association for Research in Vision and Ophthalmology's Statement for the Use of Animals in Ophthalmic and Vision Research. We used pigmented male Rex rabbits weighing 2.0–2.5 kg (Japan SLC Co. Ltd., Shizuoka, Japan). Anesthesia was carried out with intramuscular injections of a mixture of xylazine hydrochloride (5 mg/kg) and ketamine hydrochloride (3.5 mg/kg) before laser.

After laser for both eyes of 36 rabbits, we randomly divided the rabbits into three groups: the Conv 500s group (500 shots applied in the conventional setting), the SPL 500s group (500 shots in the short-pulse setting), and the SPL 1000s group (1000 shots in the short-pulse setting). In each group, 4 rabbits were used and sacrificed at 1, 7, and 14 days. Four rabbits that did not undergo photocoagulation were used as the sample of day 0, and a total of 40 rabbits were used.

#### 3.1.1. Retinal Photocoagulation

Laser photocoagulation was performed on each group using the PASCAL photocoagulator in yellow wavelength (577 nm) and Super Quad 160 contact lens. In the Conv 500s group, the laser settings were as follows: power of 200 mW, spot size of 200 *μ*m, and duration of exposure at 200 ms, with a space between each of the coagulation spot parts adjusted to approximately one coagulation spot by a single-spot process in both eyes. In the SPL 500s group, SPL photocoagulation was performed using power of 300 mW, spot size of 200 *μ*m, duration of 10 ms, and total spot number was 500. In the SPL 1000s group, PASCAL photocoagulation was performed with the same settings, except the total number of spots was 1000. The laser setting was shown in Table 1.

#### 3.1.2. Measurement of Cytokine Levels in the Vitreous Body

We measured the levels of VEGF, MCP-1, IL-6, and ICAM-1 in vitreous body at 1,7, and 14 days after laser, following the methods of Arimura et al. [17] In short, enucleated eyes were stored at −20°C for 2 days, then vitreous was obtained by removal of other tissue in the frozen condition. Samples were centrifuged for 10 minutes at 3000 ×g, then the supernatant was collected, and aliquots were kept at −20°C until the measurement. Measurements of cytokine levels were carried out with a sandwich enzyme-linked immunosorbent assay using a commercially available kit (VEGF and IL-6; Cusabio Biotech Co. Ltd., Hubei, China, MCP-1; NEO Group Inc., MA, USA, ICAM-1; Cloud-Clone Corp., TX, USA) in accordance with the manufacturer's protocol. In order to determine the protein concentration of aqueous humor, the RC DC™ protein assay kit II (Bio-Rad Laboratories, Hercules, CA, USA) was used.

### 3.2. Statistical Analyses

We carried out statistical analyses using JMP (SAS institute Inc., Tokyo, Japan). We used Bartlett's test to examine equal variances across samples, and then the statistical significances between the groups were assessed by Mann–Whitney test. Statistical analyses were performed with the Wilcoxon signed-rank test (pretreatment and posttreatment data in the same group). Values were expressed as means ± standard deviation. Differences were considered statistically significant at *p* < 0.05.

## 4. Results

### 4.1. Alteration of Central Retinal Thickness and Anterior Flare Intensity after PRP

**Figure 1:**
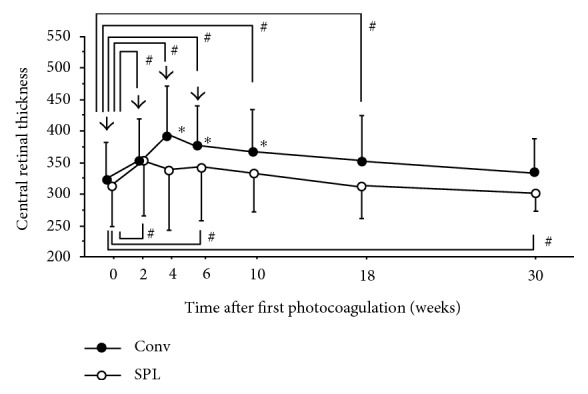
Changes in central retinal thickness (CRT) after four sessions of panretinal photocoagulation (PRP). Data represent mean ± standard deviation (SD). Arrow indicates the timing of PRP. #*p* < 0.05 (compared with CRT at PRP initiation), ^∗^*p* < 0.05 (Conv group versus SPL group).

Forty-two eyes of 42 patients (23 male, 19 female) were included in this study (the Conv group had 12 males/9 females, and the SPL group had 11 males/10 females). Basic patient characteristics are provided in Table 2. Before PRP initiation, there was no statistically significant difference between the two groups (*p* = 0.903). The mean power of laser was 257 ± 83 mW and 413 ± 107 mW (*P* < 0.0001), and the number of spots were 1203 ± 59 and 2925 ± 125 (*p* < 0.0001) in the Conv group and SPL group, respectively (Table 3). After PRP initiation, CRT in the Conv group significantly increased at 2 weeks (*p* = 0.017) and 4 weeks (*p* = 0.0013), and peaked at 6 weeks (*p* = 0.0012), then decreased but still higher at 10 weeks (*p* = 0.0015) and 18 weeks (*p* = 0.018) (Figure 1). On the other hand, the significant increase was only observed at 2 weeks (*p* = 0.0008) and 6 weeks (*p* = 0.036) in the SPL group. The significant decrease of CRT was observed at 30 weeks after PRP (*p* = 0.042). The CRT of the Conv group was significantly higher than that of the SPL group at 4 weeks (*p* = 0.029), 6 weeks (*p* = 0.047), and 10 weeks (*p* = 0.045). The percentage of eyes that showed more than 20% increase of CRT compared to baseline was 23.8% (5/21 eyes) and 9.5% (2/21 eyes) at 18 weeks, and 14.3% (3/21 eyes) and 4.8% (1/21 eyes) at 30 weeks in the Conv group and the SPL group, respectively.

**Figure 2:**
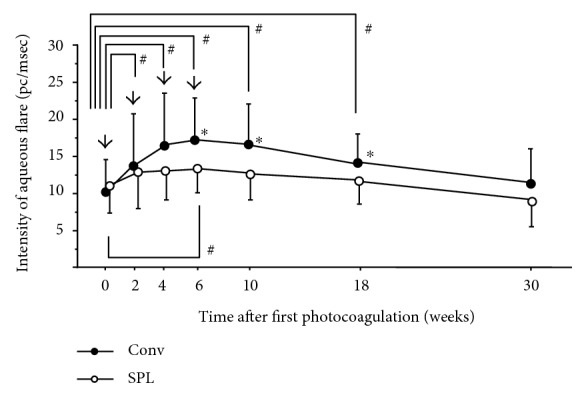
Changes in anterior flare intensity (AFI) after four sessions of panretinal photocoagulation (PRP). Data represent mean ± standard deviation (SD). Arrow indicates the timing of PRP. #*p* < 0.05 (compared with AFI at PRP initiation), ^∗^*p* < 0.05 (Conv group versus SPL group).

To estimate the degree of anterior inflammation after PRP, we also measured AFI at the same time points (Figure 2). At the time of initiation of PRP (0 weeks), there was no statistically significant difference between the two groups (*p* = 0.57). AFI significantly increased at 2 weeks (*p* = 0.0014), 4 weeks (*p* = 0.0003), 6 weeks (*p* = 0.0001), and 10 weeks (*p* = 0.0015), then decreased at 18 weeks (*p* = 0.0015), and reached to insignificant levels at 30 weeks (*p* = 0.063) in the Conv group. On the other hand, in the SPL group, significant increase of AFI after PRP was noticed only at 6 weeks (*p* = 0.0276) within the observational period. AFI in the Conv group was significantly higher than that in the SPL group at 6 weeks (*p* = 0.013), 10 weeks (*p* = 0.01), and 18 weeks (*p* = 0.0029).

The BCVA (logMAR) was 0.21 ± 0.17 and 0.19 ± 0.14 at initial stage, 0.24 ± 0.23 and 0.22 ± 0.18 at 6 weeks, 0.24 ± 0.18 and 0.21 ± 0.18 at 10 weeks, 0.28 ± 0.21 and 0.25 ± 0.17 at 18 weeks, and 0.24 ± 0.19 and 0.22 ± 0.16 at 30 weeks in the Conv group and SPL group, respectively. There was no significant difference between the groups at any time point.

Through the observational periods, no onsets of NVI and NVG occurred; however, 1 case of vitreous hemorrhage was noticed in each group (the Conv group: at 29 weeks after PRP, the SPL group: at 30 weeks after PRP) (Table 3). In these cases, the dense of hemorrhage was not severe and absorbed within 1 month, and thus vitrectomy was not carried out. No intravitreal injection of anti-VEGF drug or steroids was carried out in both groups after PRP. Since the new onset of NVE was noticed, the additional laser treatment was carried out in 2 (9.5%) and 3 (14.3%) cases in the Conv group and the SPL group, respectively. Chi-square test showed that there were no significant differences between the Conv group and the SPL group in the ratio of vitreous hemorrhage and the number of the additional laser treatment.

### 4.2. PRP Induced Intravitreal Concentration of Cytokines in Rabbits

**Figure 3:**
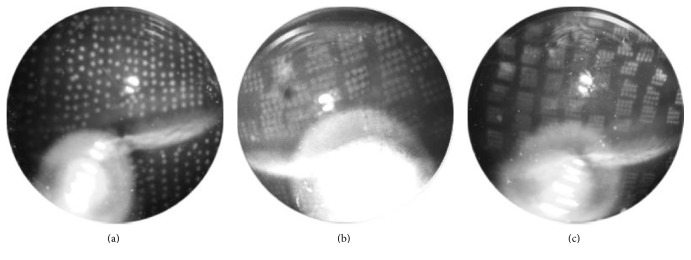
Fundus photography of the eye of a rabbit immediately after retinal laser photocoagulation. (a) 500 spots applied by the conventional argon laser. (b) 500 spots applied by short-pulse laser (SPL). (c) 1000 spots applied by SPL.

As described in the Methods section, we performed PRP 500 shots in the eyes of the pigmented rabbits in the Conv 500s group using the conventional argon laser, 500 shots in the SPL 500s group, and 1000 shots in the SPL 1000s group using PASCAL photocoagulator (Figure 3).

**Figure 4:**
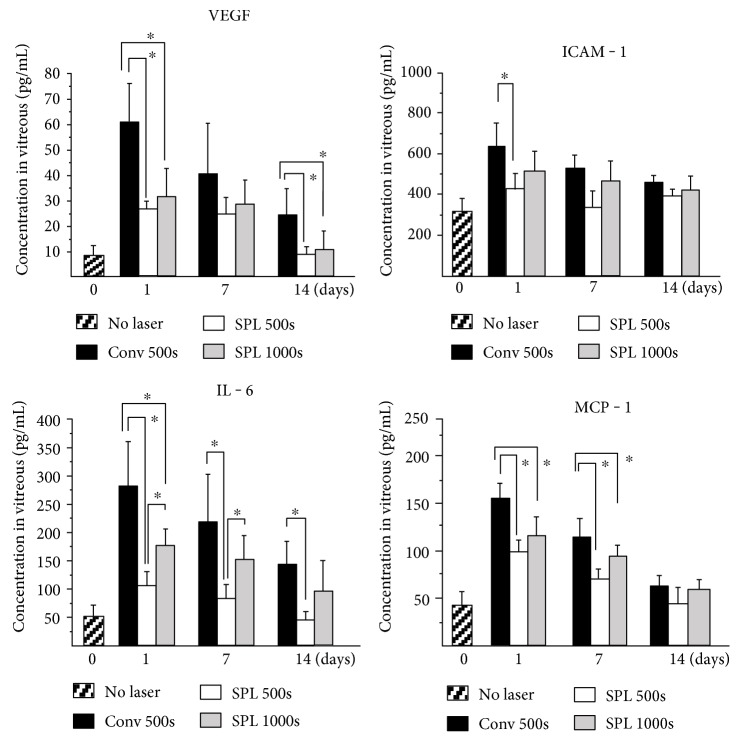
Alteration in the vitreous levels of inflammatory and angiogenic cytokines after panretinal photocoagulation (PRP) using the conventional laser with 500 spots (Conv 500s), short-pulse laser (SPL) with 500 spots (SPL 500), and SPL with 1000 spots (SPL 1000s). Concentrations of VEGF, IL-6, ICAM-1, and MCP-1 in the vitreous body were measured before and on 1, 7, and 14 days after PRP. Statistically significant difference as compared to the Conv group (^∗^*p* < 0.05). Vertical bars indicate standard deviation. (*n* = 8).

Figure 4 shows the temporal profiles of the amounts of VEGF, IL-6, ICAM-1, and MCP-1 in the vitreous body. VEGF levels in the Conv 500s group increased from a baseline (before PRP) of 7.3 ± 3.9 pg/mL to 59.7 ± 14.1 pg/mL, then decreased to 41.7 ± 18.2 pg/mL after 7 days and 24.5 ± 10.5 pg/mL after 14 days. In the eyes of the SPL 500s group and SPL 1000s group, VEGF levels were significantly lower at day 1 (*p* = 0.0039 and *p* = 0.011, resp.) and at day 14 (*p* = 0.025, and *p* = 0.037, resp.) than in the eyes of the Conv 500s group. There was no significant difference in VEGF levels between the SPL 500s and SPL 1000s group at any time point.

IL-6 levels in the vitreous body transiently elevated at day 1. IL-6 levels in SPL 500s group were significantly lower at day 1 (*p* = 0.0065), day 7 (*p* = 0.016), and day 14 (*p* = 0.0039) than those in the Conv 500s group, whereas the significant difference between the Conv 500s group and the SPL 1000s group was noticed only at day 1 (*p* = 0.048). IL-6 levels in SPL 500s were significantly lower than those in SPL 1000s group at day 1 (*p* = 0.0039) and day 7 (*p* = 0.0041).

ICAM-1 levels in the vitreous body increased at day 1 and gradually decreased thereafter. ICAM-1 levels in the SPL 500s group were significantly lower than those in the Conv 500s group (*p* = 0.0039) on day 1.

MCP-1 levels in the Conv 500s group dramatically increased and then decreased. In the SPL 500s group and SPL 1000s group, the significantly lower levels were observed at day 1 (*p* = 0.01 and *p* = 0.046, resp.), day 7 (*p* = 0.01 and *p* = 0.0163, resp.), and day 14 (*p* = 0.0065) than those in the Conv 500s group.

**Figure 5:**
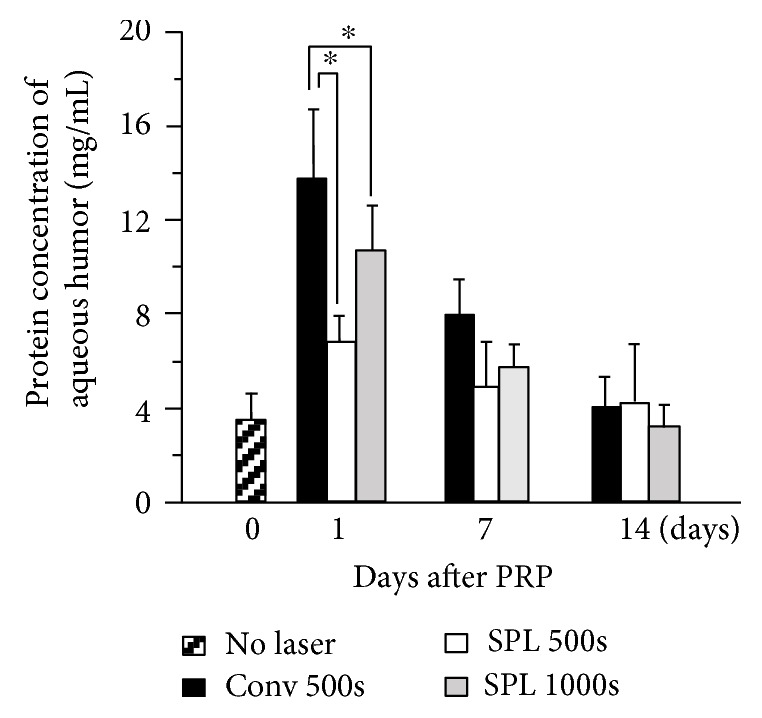
Protein concentration changes in the aqueous humor after panretinal photocoagulation (PRP) using conventional laser with 500 spots (Conv 500), short-pulse laser (SPL) with 500 spots (SPL 500), and SPL with 1000 spots (SPL 1000s). Statistically significant difference as compared to the Conv group (^∗^*p* < 0.05). Vertical bars indicate standard deviation (*n* = 8).

The mean levels of aqueous proteins increased from 3.7 ± 1.1 to 14.6 ± 3.5 at day 1, followed by a decrease to 8.6 ± 2.1 at day 7 and 4.1 ± 1.7 at day 14 (Figure 5). At day 1, the levels in SPL 500s and SPL 1000s groups were significantly lower (*p* = 0.0016 and *p* = 0.038, resp.) than those in the Conv 500s group. There was no significant difference among the groups at day 7 and day 14.

## 5. Discussion

Muqit et al. showed that PDR eyes treated with a single 20 ms session of PRP using PASCAL demonstrated less increased total macular thickness after treatment, compared with conventional 100 ms single spot delivered over multiple sessions [14]. In their study, the total number of spots was the same, 1500 shots in each group, indicating that SPL contributes to prevent the worsening of DME in the same shot number. However, it is recognized that SPL requires a greater number of spots because the expansion of scars is less than that is required with traditional laser. The increased number of laser spots may enhance the risks such as increased inflammation and the worsening of DME. Therefore, we were interested to know whether SPL is a less-invasive treatment than traditional lasers, even if double the number of shots were performed for the management of PDR. In our data, the total number of spots in the SPL group was 2.4 times more than that in the Conv group. Nevertheless, the eyes that received laser with shorter duration showed significantly less increase in CRT than eyes that received conventional laser. Similar to our results, Mirshahi et al. reported significantly smaller increase in CRT from baseline in 20 ms SPL group than in the conventional laser group [18]. In their study, the number of spots in the SPL group was approximately 1.4 times more than that in the conventional laser group [18]. Even if the number of spots was greater, SPL is able to inhibit the worsening of DME after PRP in the patients with diabetic retinopathy.

The effect of photocoagulation can be determined by three interdependent parameters, including spot size, power, and pulse duration. In our clinical study, the average power intensity was 257 mW and 414 mW, and the pulse duration was 200 ms and 20 ms in the Conv and SPL groups, respectively. Thus, the energy per spot was approximately 6.2 times higher in the Conv group than in the SPL group. Although the number of laser spots in SPL group was greater than that in the Conv group, the total energy to which the eye was exposed in the Conv group (61.8 J) was 2.5 times higher than that in the SPL group (24.3 J). The higher energy of lasers probably results in the greater progression of AFI and macular thickening in the patients with diabetes in the Conv group.

Our clinical data showed that conventional laser induced a significant increase in aqueous flare after PRP, indicating a breakdown of the blood-aqueous barrier (BAB) after laser treatment, which is consistent with the previous reports [19–21]. On the other hand, AFI in the SPL group tended to increase but the difference was not statistically significant. Moreover, the photocoagulated eyes of rabbits showed that the protein concentration in the aqueous humor in the SPL group was less than that in the Conv group. These data suggest the usage of SPL is useful in preventing the breakdown of BAB. The increase in protein levels was transient in the rabbit eyes, whereas high AFI levels lasted until ≥10 weeks after PRP initiation in patients with diabetes. This difference is probably because PRP was repeated four times every 2 weeks, which may have led to persistent anterior inflammation in the patients with diabetic retinopathy. In addition, it is probable that the ability to recover from the breakdown of BAB is greater in the eyes of normal rabbits than in the eyes of diabetic patients with retinal ischemia.

We investigated the effect on inflammatory cytokine levels after laser burns, depending on the energy and the number of spots using normal pigmented rabbits. Our data showed that SPL induced less expression of VEGF, IL-6, MCP-1, and ICAM-1 than the conventional laser treatment. This finding is similar to the report by Ito et al., in which the lower expression of inflammatory cytokines in the eyes treated with SPL was noticed in the sensory retina and choroid of mice [7]. It is probable that the greater intravitreal cytokine levels resulted from the enhanced release from retina and choroid after lasers. The increase in the number of laser spots may lead to further inflammation and the increase in cytokine levels in vitreous. Actually, our animal data showed that AFI 1 day (500 spots) was significantly higher in the conventional laser group than in the SPL 500s group, but no significant difference was noticed compared with that in the SPL 1000s group. However, the cytokines which showed the significantly higher amount in the SPL 1000s group than in the SPL 500s group were only IL-6, which is capable to promote the angiogenesis and vascular permeability [9, 22, 23]. It is possible that the IL-6 expression levels are influenced by the number of laser spots, which may be related to the pathogenesis in the breakdown of BAB after lasers.

VEGF promotes the neovascularization and a vascular permeability resulting in macular edema [24, 25]. Clinically, PRP has two opposing effects, namely suppression of proliferative change and transient worsening of the macula edema, both of which are related to the intravitreal VEGF levels [7, 8, 26, 27]. Itaya et al. showed that MCP-1 may contribute to the upregulation of VEGF after photocoagulation [8]. VEGF and MCP-1 levels are reported to be increased in the vitreous of patients with DME, indicating the association with the pathogenesis of DME [9]. Our data in this study showed that the usage of SPL, either 500 or 1000 shots, decreased laser-induced expression of both MCP-1 and VEGF in comparison to the conventional lasers. Taken together, the suppression of MCP-1 levels in the eyes that underwent SPL may contribute to the decreased VEGF levels in the vitreous. ICAM-1 is an intracellular adhesion molecule necessary for the adhesion of leukocytes to capillary endothelium [28]. The reduction of ICAM-1 level by using SPL may contribute to the inhibition of leukocyte recruitment and vascular permeability in comparison to the conventional lasers.

Recently, we showed that the PRP-induced increase in VEGF and proinflammatory cytokine levels in aqueous and vitreous humors could be decreased by the administration of triamcinolone via intravitreal or sub-Tenon injection in the eyes of pigmented rabbits [17]. Moreover, many clinical studies demonstrated that the administration of steroids is effective for preventing the progression of DME after PRP [29–32]. These data indicate the efficacy of steroids as anti-inflammatory drugs; however, steroid use is associated with side effects, such as elevated intraocular pressure and cataract progression. SPL, which enables us to perform less-invasive laser treatment, would be a fundamental measure to decrease the inflammatory reaction induced by PRP.

On the basis of our data, it seems that the progression of DME and ocular inflammation after PRP is transient and that it spontaneously recovers within 6 months. However, we should pay attention to the fact that 23.8% and 14.3% of eyes that underwent conventional argon laser treatment showed persistent macular edema at 18 and 30 weeks after PRP, respectively, whereas only 9.5% and 4.8% did in the SPL group. Although our data indicate that SPL is a less-invasive tool for the management of PDR and DME, it is also clinically important that sufficient application of lasers for the ischemic areas is required to prevent proliferative changes. In our case series, the onset of NVI and NVG was not observed, and no significant differences were not noticed in the ratio of vitreous hemorrhage and the number of the additional laser treatment between the Conv group and the SPL group during the follow-up period. However, the sample size is too small and the observational periods are too short to determine whether SPL is more efficacious in preventing further future recurrence or persistence of neovascularization.

## 6. Conclusion

Our clinical and experimental studies demonstrated that SPL is an effective tool to prevent the worsening of DME and AFI even if the total number of spots was more than twice compared with conventional laser. The increase in spot number affected IL-6 expression levels in the vitreous, but not those in VEGF, ICAM-1, and MCP-1, indicating an association between the pathogenesis of the temporal progression of DME and AFI, and the dose dependency of SPL treatment.

## Figures and Tables

**Table 1:** Laser parameter settings.

	Conv 500s	SPL 500s	SPL 1000s
Power (mW)	200	300	300
Pulse duration (ms)	200	20	20
Spot size (um)	200	200	200
Wavelength (nm)	532	532	532
Number of spots	500	500	1000
Total energy/spot (mJ)	40	6	6
Total energy/eye (J)	20	3	6

Conv: conventional; SPL: short-pulse laser.

**Table 2:** Baseline characteristics at the time of registration.

	Conv group^a^ (*n* = 21)	SPL group^b^ (*n* = 21)	*p* value
Age (years)	68.2 ± 7.2	66.3 ± 9.5	.43^a^
Gender (male/female)	12/9	11/10	.65^b^
Duration of DM (years)	12.5 ± 4.4	13.1 ± 5.9	.38^a^
Hemoglobin A1c (%)	7.3 ± 0.9	7.1 ± 1.1	.51^a^
Insulin therapy	9 (42.9%)	10 (47.6%)	.34^b^
Left eye: right eye	8 : 11	7 : 14	.52^b^
Phakic eyes	9 (42.9%)	10 (47.6%)	.51^b^
The presence of diabetic macular edema (%)	16 (76.1%)	17 (81.0%)	.73^b^
Focal	8	9	
Diffuse	8	8	

^a^Mann–Whitney test. ^b^Chi-square test. DM: diabetes mellitus; Conv: conventional; SPL: short-pulse laser.

**Table 3:** Summary of parameters relating to lasers.

	Conv group	SPL group
Power (mW)	257 ± 83	413 ± 107
Pulse duration (ms)	200	20
Spot size (um)	200	200
Wavelength (nm)	532	532
Total energy/spot (mJ)	51.4 ± 16.6	8.3 ± 10.4
Number of spots	1203 ± 59	2925 ± 125
Type of laser spots	Single spot	Pattern spot (5 × 5, 4 × 4 arrays)
Complications		
Vitreous hemorrhage (%)	1 (4.8)	1 (4.8)
Neovascular glaucoma (%)	0 (0)	0 (0)

Conv: conventional; SPL: short-pulse laser.

## References

[1] The Diabetic Retinopathy Study Research Group (1981). Photocoagulation treatment of proliferative diabetic retinopathy. Clinical application of diabetic retinopathy study (DRS) findings, DRS report number 8. *Ophthalmology*.

[2] Branch Vein Occlusion Study Group (1986). Argon laser scatter photocoagulation for prevention of neovascularization and vitreous hemorrhage in branch vein occlusion. A randomized clinical trial. *Archives of Ophthalmology*.

[3] Paulus Y. M., Jain A., Gariano R. F. (2008). Healing of retinal photocoagulation lesions. *Investigative Ophthalmology & Visual Science*.

[4] Takahashi A., Nagaoka T., Sato E., Yoshida A. (2008). Effect of panretinal photocoagulation on choroidal circulation in the foveal region in patients with severe diabetic retinopathy. *The British Journal of Ophthalmology*.

[5] Oh I. K., Kim S. W., Oh J., Lee T. S., Huh K. (2010). Inflammatory and angiogenic factors in the aqueous humor and the relationship to diabetic retinopathy. *Current Eye Research*.

[6] Shimura M., Yasuda K., Nakazawa T. (2009). Panretinal photocoagulation induces pro-inflammatory cytokines and macular thickening in high-risk proliferative diabetic retinopathy. *Graefe's Archive for Clinical and Experimental Ophthalmology*.

[7] Ito A., Hirano Y., Nozaki M., Ashikari M., Sugitani K., Ogura Y. (2015). Short pulse laser induces less inflammatory cytokines in the murine retina after laser photocoagulation. *Ophthalmic Research*.

[8] Itaya M., Sakurai E., Nozaki M. (2007). Upregulation of VEGF in murine retina via monocyte recruitment after retinal scatter laser photocoagulation. *Investigative Ophthalmology & Visual Science*.

[9] Funatsu H., Noma H., Mimura T., Eguchi S., Hori S. (2009). Association of vitreous inflammatory factors with diabetic macular edema. *Ophthalmology*.

[10] Sanghvi C., McLauchlan R., Delgado C. (2008). Initial experience with the Pascal photocoagulator: a pilot study of 75 procedures. *The British Journal of Ophthalmology*.

[11] Jain A., Blumenkranz M. S., Paulus Y. (2008). Effect of pulse duration on size and character of the lesion in retinal photocoagulation. *Archives of ophthalmology (Chicago, Ill.: 1960)*.

[12] Muqit M. M. K., Gray J. C. B., Marcellino G. R. (2010). In vivo laser-tissue interactions and healing responses from 20- vs 100-millisecond pulse Pascal photocoagulation burns. *Archives of ophthalmology (Chicago, Ill.: 1960)*.

[13] Muqit M. M. K., Marcellino G. R., Gray J. C. B. (2010). Pain responses of Pascal 20 ms multi-spot and 100 ms single-spot panretinal photocoagulation: Manchester Pascal Study, MAPASS report 2. *The British Journal of Ophthalmology*.

[14] Muqit M. M. K., Marcellino G. R., Henson D. B., Fenerty C. H., Stanga P. E. (2011). Randomized clinical trial to evaluate the effects of Pascal panretinal photocoagulation on macular nerve fiber layer: Manchester Pascal Study report 3. *Retina*.

[15] Muqit M. M. K., Marcellino G. R., Henson D. B., Young L. B., Turner G. S., Stanga P. E. (2011). Pascal panretinal laser ablation and regression analysis in proliferative diabetic retinopathy: Manchester Pascal Study Report 4. *Eye*.

[16] Chappelow A. V., Tan K., Waheed N. K., Kaiser P. K. (2012). Panretinal photocoagulation for proliferative diabetic retinopathy: pattern scan laser versus argon laser. *American Journal of Ophthalmology*.

[17] Arimura S., Takamura Y., Miyake S. (2016). The effect of triamcinolone acetonide or bevacizumab on the levels of proinflammatory cytokines after retinal laser photocoagulation in pigmented rabbits. *Experimental Eye Research*.

[18] Mirshahi A., Lashay A., Roozbahani M. (2013). Pain score of patients undergoing single spot, short pulse laser versus conventional laser for diabetic retinopathy. *Graefe’s Archive for Clinical and Experimental Ophthalmology*.

[19] Larsson L. I., Nuija E. (2001). Increased permeability of the blood-aqueous barrier after panretinal photocoagulation for proliferative diabetic retinopathy. *Acta Ophthalmologica Scandinavica*.

[20] Moriarty A. P., Spalton D. J., Shilling J. S., Ffytche T. J., Bulsara M. (1996). Breakdown of the blood-aqueous barrier after argon laser panretinal photocoagulation for proliferative diabetic retinopathy. *Ophthalmology*.

[21] Suto C., Hori S., Kato S. (2008). Management of type 2 diabetics requiring panretinal photocoagulation and cataract surgery. *Journal of Cataract and Refractive Surgery*.

[22] Wei L. H., Kuo M. L., Chen C. A. (2003). Interleukin-6 promotes cervical tumor growth by VEGF-dependent angiogenesis via a STAT3 pathway. *Oncogene*.

[23] Funatsu H., Yamashita H., Ikeda T., Mimura T., Eguchi S., Hori S. (2003). Vitreous levels of interleukin-6 and vascular endothelial growth factor are related to diabetic macular edema. *Ophthalmology*.

[24] Dvorak H. F., Brown L. F., Detmar M., Dvorak A. M. (1995). Vascular permeability factor/vascular endothelial growth factor, microvascular hyperpermeability, and angiogenesis. *The American Journal of Pathology*.

[25] Keck P. J., Hauser S. D., Krivi G. (1989). Vascular permeability factor, an endothelial cell mitogen related to PDGF. *Science*.

[26] (1985). Photocoagulation for diabetic macular edema. Early treatment diabetic retinopathy study report number 1. Early treatment diabetic retinopathy study research group. *Archives of Ophthalmology*.

[27] Boulton M., Foreman D., Williams G., McLeod D. (1998). VEGF localisation in diabetic retinopathy. *The British Journal of Ophthalmology*.

[28] Tamura H., Miyamoto K., Kiryu J. (2005). Intravitreal injection of corticosteroid attenuates leukostasis and vascular leakage in experimental diabetic retina. *Investigative Ophthalmology & Visual Science*.

[29] Cho W. B., Moon J. W., Kim H. C. (2010). Intravitreal triamcinolone and bevacizumab as adjunctive treatments to panretinal photocoagulation in diabetic retinopathy. *The British Journal of Ophthalmology*.

[30] Shimura M., Yasuda K., Shiono T. (2006). Posterior sub-Tenon’s capsule injection of triamcinolone acetonide prevents panretinal photocoagulation-induced visual dysfunction in patients with severe diabetic retinopathy and good vision. *Ophthalmology*.

[31] Unoki N., Nishijima K., Kita M. (2009). Randomised controlled trial of posterior sub-Tenon triamcinolone as adjunct to panretinal photocoagulation for treatment of diabetic retinopathy. *The British Journal of Ophthalmology*.

[32] Mirshahi A., Shenazandi H., Lashay A., Faghihi H., Alimahmoudi A., Dianat S. (2010). Intravitreal triamcinolone as an adjunct to standard laser therapy in coexisting high-risk proliferative diabetic retinopathy and clinically significant macular edema. *Retina*.

